# Supplementary figures and images for: Trace elements during primordial plexiform network formation in human cerebral organoids
Source: PeerJ. 2017 Feb 8;5:e2927. doi: 10.7717/peerj.2927 (PMC5301978; doi:10.7717/peerj.2927)

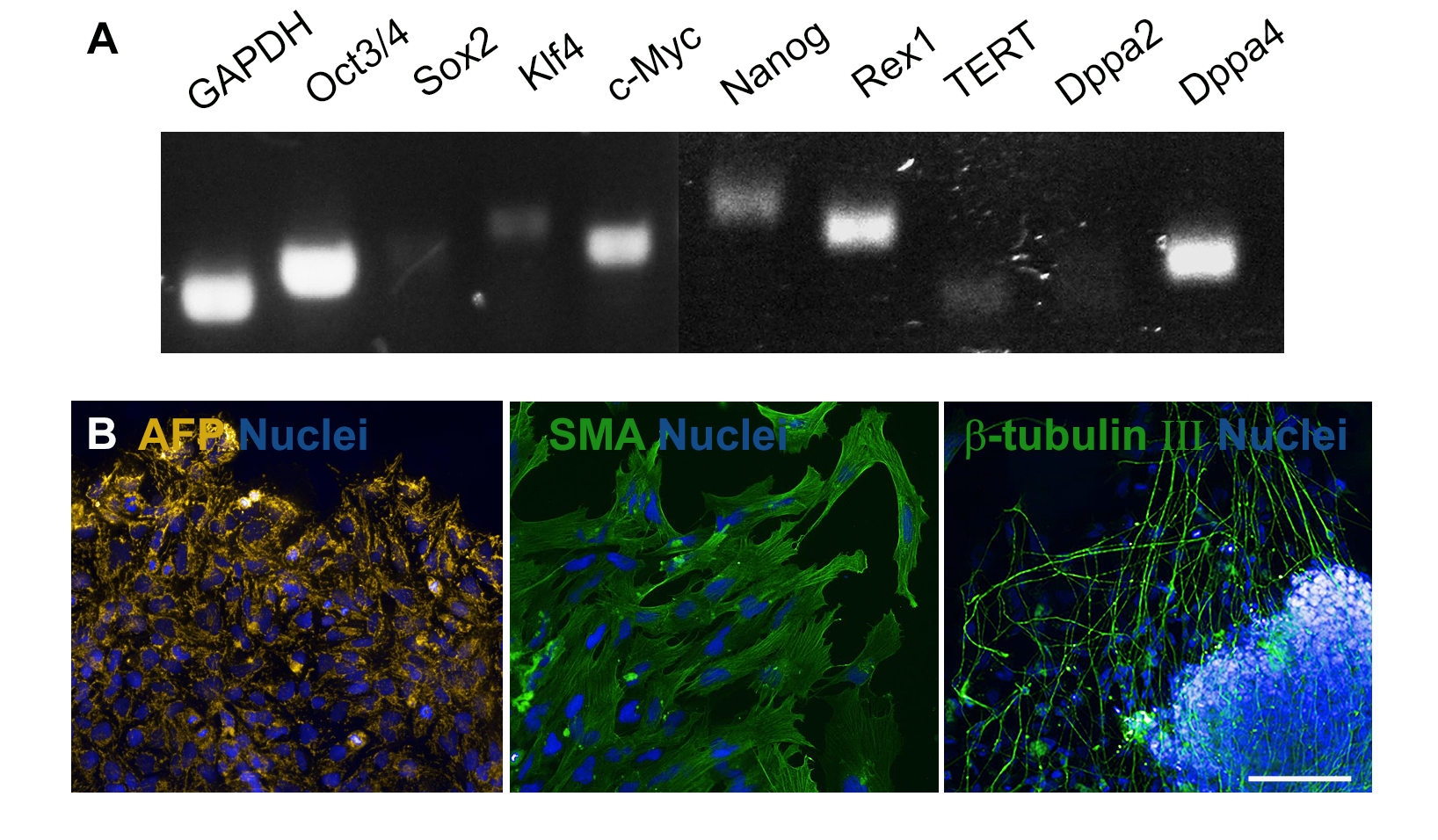

Supplement: Figure S1 — (A) RT-PCR analysis for pluripotency markers. GAPDH was used as loading control. (B) Differentiation of iPS cells into cells derived from endoderm (alpha-fetoprotein, AFP), mesoderm (alpha smooth muscle actin, SMA) and ectoderm (β-tubulin III) in embryoid bodies assay. Scale bars: 100 µm. [file peerj-05-2927-s001.png]

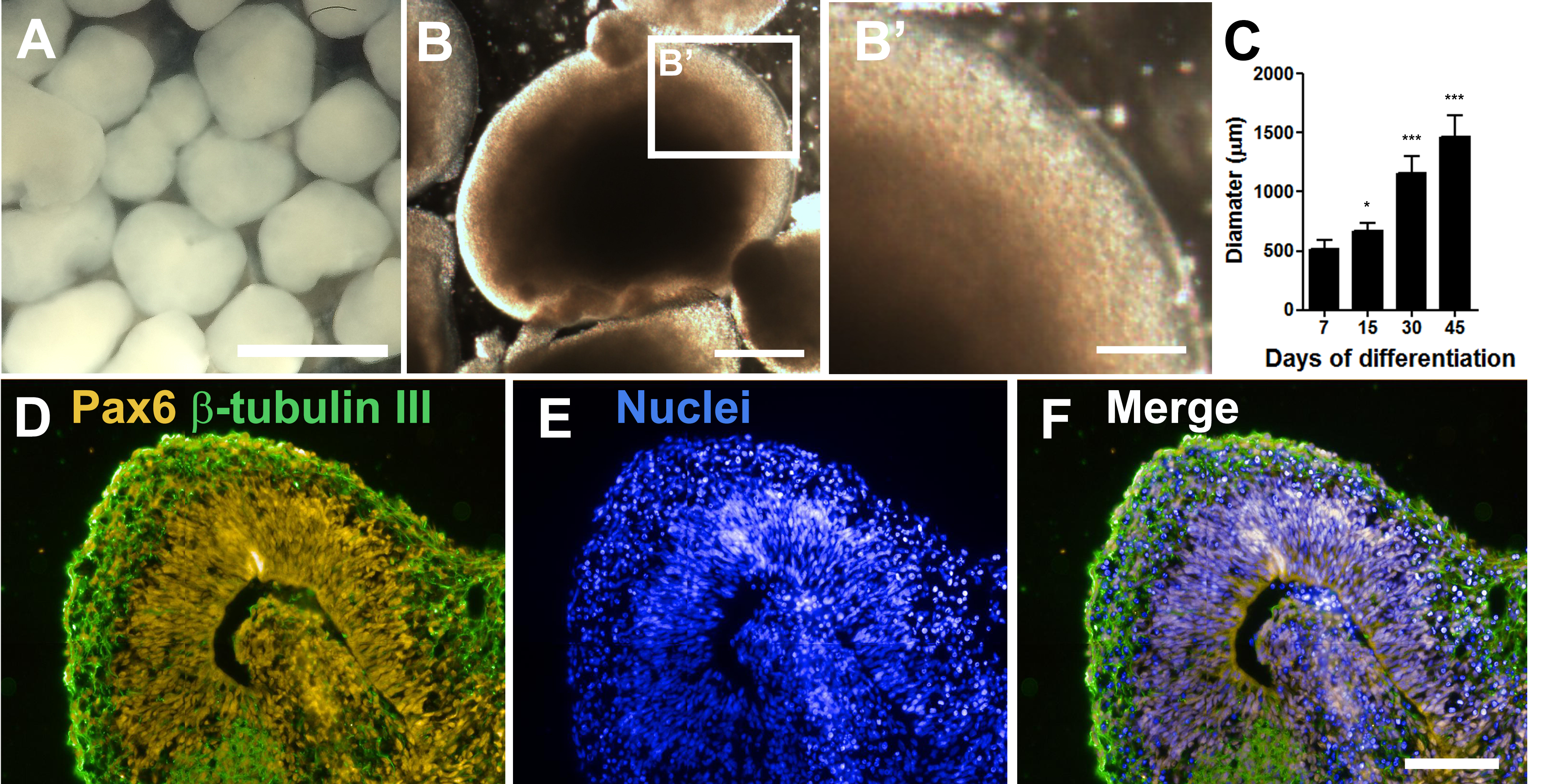

Supplement: Figure S2 — (A) Cerebral organoids of 45-days old produced from iPS cells derived from skin fibroblasts present similar coloring and texture to hESC-derived cerebral organoids. (B and B’) A 30-days old organoid in detail showing different hues according to different cell layers. (C) Along differentiation, organoids’ diameter doubled between days 7 and 30 in culture and tripled after 45 days. (D) Organoid section stained for β-tubulin III and PAX6 to show distintic neuronal and neural progenitor cells distribution, respectively. (E) DAPI stained nuclei. (F) Merged channels. The graph represents mean ±S.D. n = 8 for 7-days old organoids, n = 15 for 15-days old organoids, n = 7 for 30-days old organoids, n = 10 for 45-days old organoids. P < 0.05 for 7-days old versus 30 and 45-days old organoids, for 15-days old organoids versus 30 and 45-days old organoids, and for 30-days old organoids versus 45-days old organoids. Cerebral organoids were obtained from one assay. Scale bars: A = 1.5 mm, B = 250 µm, B’ = 75 µm, D–F = 100 µm. [file peerj-05-2927-s002.png]
