# Supplementary material for: Trace elements during primordial plexiform network formation in human cerebral organoids
Source: PeerJ. 2017 Feb 8;5:e2927. doi: 10.7717/peerj.2927 (PMC5301978; doi:10.7717/peerj.2927)
Supplement: Table S1 — Cerebral organoid differentiation media were analyzed by SR-XRF in order to assess background signals generated by elements present in culture media (EB medium, neuroinduction medium and neurodifferentiation medium). Values are shown as percentage of total values found in cerebral organoids. Elements Fe and Zn were not detected (ND) within XRF range. [file peerj-05-2927-s003.docx]

| **Cell differentiation media elements** | ***30-days old organoids* (%)** | ***45-days old organoids* (%)** |
| --- | --- | --- |
| **P** | 0.000173 | 0.000244 |
| **S** | 0.004192 | 0.004507 |
| **K** | 0.002124 | 0.003181 |
| **Ca** | 0.024248 | 0.031343 |
| **Fe** | ND | ND |
| **Zn** | ND | ND |
