# Supplementary material for: Trace elements during primordial plexiform network formation in human cerebral organoids
Source: PeerJ. 2017 Feb 8;5:e2927. doi: 10.7717/peerj.2927 (PMC5301978; doi:10.7717/peerj.2927)
Supplement: Table S2 — Matrigel was used during organoid formation; therefore, it was analyzed by SR-XRF in order to assess background signals generated by this reagent. Values are shown as percentage of total values found in organoids. Elements Fe and Zn were not detected (ND) within XRF range. [file peerj-05-2927-s004.docx]

| **Matrigel elements** | ***30-days old organoids* (%)** | ***45-days old organoids* (%)** |
| --- | --- | --- |
| **P** | 1.78*10^-13^ | 2.51*10^-13^ |
| **S** | 3.38*10^-12^ | 3.63*10^-12^ |
| **K** | 4.11*10^-12^ | 6.15*10^-12^ |
| **Ca** | 3.01*10^-11^ | 3.89*10^-11^ |
| **Fe** | ND | ND |
| **Zn** | ND | ND |
