# Supplementary material for: Trace elements during primordial plexiform network formation in human cerebral organoids
Source: PeerJ. 2017 Feb 8;5:e2927. doi: 10.7717/peerj.2927 (PMC5301978; doi:10.7717/peerj.2927)
Supplement: Table S3 — Values from Rajan et al. (1997) are an average from different regions of cerebrum cortex and S.D. refers to the number of different regions assessed. [file peerj-05-2927-s005.docx]

| **Concentration (ppm)** | | | | |
| --- | --- | --- | --- | --- |
| **Element** | Sofic et al.  (1988) | Dexter et al.  (1991) | Rajan et al.  (1997) | Rahil-Khazen et al. (2002) |
| **P** |  |  | 1,920±256 |  |
| **S** |  |  |  |  |
| **K** |  |  | 3,180±306 |  |
| **Ca** |  |  | 175±6 |  |
| **Fe** | 28±5.4 | 58.9 | 49±7.5 | 50.2 |
| **Zn** |  | 31.25±3.0 | 12.7±6 | 11.8 |
