# Supplementary material for: Trace elements during primordial plexiform network formation in human cerebral organoids
Source: PeerJ. 2017 Feb 8;5:e2927. doi: 10.7717/peerj.2927 (PMC5301978; doi:10.7717/peerj.2927)
Supplement: Data S1 [file peerj-05-2927-s006.doc]

|  | **Diameter size (μm)** | | |  | |
| --- | --- | --- | --- | --- | --- |
|  | **Days of differentiation** | | | | |
| **Sample** | **7 days** | **15 days** | **30 days** | | **45 days** |
| 1 | 226 | 982 | 1,186 | | 1,300 |
| 2 | 285 | 583 | 692 | | 1,324 |
| 3 | 307 | 836 | 1,070 | | 1,435 |
| 4 | 322 | 509 | 1,225 | | 1,286 |
| 5 | 353 | 439 | 1,201 | | 1,593 |
| 6 | 275 | 467 | 703 | | 1,268 |
| 7 | 302 | 436 | 700 | | 1,652 |
| 8 | 152 | 326 | 447 | | 1,233 |
| 9 | 296 | 498 | 407 | | 1,179 |
| 10 | 220 | 387 | 512 | | 1,466 |
| 11 | 364 | 412 | 738 | | 1,568 |
| 12 | 299 | 562 | 423 | | 1,369 |
| 13 | 303 | 317 | 297 | | 901 |
| 14 | 316 | 947 | 586 | | 1,643 |
| 15 | 245 | 561 | 1,078 | | 446 |
| 16 | 247 | 889 | 704 | | 613 |
| 17 | 306 | 1,094 | 1,001 | | 606 |
| 18 | 262 | 1,224 | 706 | | 1,422 |
| 19 | 298 | 1,144 | 368 | |  |
| 20 | 442 | 808 | 250 | |  |
| 21 | 386 | 558 | 277 | |  |
| 22 | 398 | 533 | 1,243 | |  |
| 23 | 239 | 445 | 771 | |  |
| 24 | 304 | 715 | 266 | |  |
| 25 | 250 | 664 | 228 | |  |
| 26 | 531 | 363 | 250 | |  |
| 27 | 284 | 709 | 306 | |  |
| 28 | 283 | 409 | 254 | |  |
| 29 | 338 | 537 | 576 | |  |
| 30 | 333 | 440 | 1,074 | |  |
| 31 | 397 | 440 | 742 | |  |
| 32 | 292 | 374 | 380 | |  |
| 33 | 330 | 365 | 179 | |  |
| 34 | 300 | 489 | 474 | |  |
| 35 | 274 | 401 | 304 | |  |
| 36 | 367 | 459 | 413 | |  |
| 37 | 306 | 407 | 1,250 | |  |
| 38 | 345 | 669 | 1,392 | |  |
| 39 | 366 | 398 | 604 | |  |
| 40 | 231 | 392 | 400 | |  |
| 41 | 242 | 428 | 472 | |  |
| 42 | 362 | 352 | 323 | |  |
| 43 | 291 | 423 | 323 | |  |
| 44 | 307 | 399 | 374 | |  |
| 45 | 411 | 617 | 666 | |  |
| 46 | 373 | 487 | 484 | |  |
| 47 | 262 | 430 | 762 | |  |
| 48 | 306 | 438 | 1,150 | |  |
| 49 | 331 | 372 | 1,256 | |  |
| 50 | 423 | 951 | 1,498 | |  |
| 51 | 310 | 374 | 1,149 | |  |
| 52 | 385 | 589 | 1,371 | |  |
| 53 | 339 | 1,017 | 1,403 | |  |
| 54 | 285 | 724 | 1,226 | |  |
| 55 | 318 | 664 | 604 | |  |
| 56 | 411 | 719 | 694 | |  |
| 57 | 172 | 768 |  | |  |
| 58 | 310 | 462 |  | |  |
| 59 | 204 | 366 |  | |  |
| 60 | 238 | 986 |  | |  |
| 61 | 154 | 944 |  | |  |
| 62 | 176 | 1,053 |  | |  |
| 63 | 219 | 619 |  | |  |
| 64 | 249 | 607 |  | |  |
| 65 | 273 | 486 |  | |  |
| 66 | 138 | 539 |  | |  |
| 67 | 264 | 859 |  | |  |
| 68 | 216 | 834 |  | |  |
| 69 | 161 | 439 |  | |  |
| 70 | 194 | 574 |  | |  |
| 71 | 353 | 221 |  | |  |
| 72 | 507 | 366 |  | |  |
| 73 | 170 | 243 |  | |  |
| 74 | 410 | 724 |  | |  |
| 75 | 239 | 469 |  | |  |
| 76 | 248 | 581 |  | |  |
| 77 | 290 | 502 |  | |  |
| 78 | 297 | 518 |  | |  |
| 79 | 154 | 313 |  | |  |
| 80 | 397 | 661 |  | |  |
| 81 | 249 | 1,292 |  | |  |
| 82 | 386 | 650 |  | |  |
| 83 | 453 | 989 |  | |  |
| 84 | 272 | 703 |  | |  |
| 85 | 313 | 711 |  | |  |
| 86 | 365 | 795 |  | |  |
| 87 | 247 | 613 |  | |  |
| 88 | 303 | 918 |  | |  |
| 89 | 297 | 725 |  | |  |
| 90 | 285 | 885 |  | |  |
| 91 | 280 |  |  | |  |
| 92 | 214 |  |  | |  |
| 93 | 367 |  |  | |  |
| 94 | 158 |  |  | |  |
| 95 | 229 |  |  | |  |
| 96 | 231 |  |  | |  |
| 97 | 176 |  |  | |  |
| 98 | 269 |  |  | |  |
| 99 | 165 |  |  | |  |
| 100 | 328 |  |  | |  |
| 101 | 99 |  |  | |  |
| 102 | 340 |  |  | |  |
| 103 | 166 |  |  | |  |
| 104 | 279 |  |  | |  |
| 105 | 232 |  |  | |  |
| 106 | 262 |  |  | |  |
| 107 | 174 |  |  | |  |
| **Mean** | **288.8** | **606.5** | **704.1** | | **1,239** |
| **St. Deviation** | **80.2** | **236.4** | **383.9** | | **364.7** |
| **St. Error** | **7.75** | **24.91** | **51.3** | | **85.96** |
