# Supplementary material for: Trace elements during primordial plexiform network formation in human cerebral organoids
Source: PeerJ. 2017 Feb 8;5:e2927. doi: 10.7717/peerj.2927 (PMC5301978; doi:10.7717/peerj.2927)
Supplement: Data S2 [file peerj-05-2927-s007.doc]

| **Sample** | **30-days old organoids** | | | | **45-days old organoids** | | |
| --- | --- | --- | --- | --- | --- | --- | --- |
|  | **Ventricle area (mm^2^)** | | **Organoid slice area (mm^2^)** | **Ventricle area/ organoid slice area** | **Ventricle area (mm^2^)** | **Organoid slice area (mm^2^)** | **Ventricle area/ organoid slice area** |
| 1 | 0.01 | | 2.5 | 0.4 | 0.01 | 0.32 | 3.1 |
| 2 | 0.034 | | 2.5 | 1.4 | 0.004 | 0.32 | 1.2 |
| 3 | 0.004 | | 0.99 | 0.4 | 0.009 | 1.33 | 0.7 |
| 4 | 0.004 | | 0.99 | 0.4 | 0.018 | 1.33 | 1.4 |
| 5 | 0.009 | | 0.69 | 1.3 | 0.015 | 1.33 | 1.1 |
| 6 | 0.002 | | 0.69 | 0.3 | 0.018 | 1.11 | 1.6 |
| 7 | 0.007 | | 0.69 | 1.0 | 0.038 | 1.24 | 3.1 |
| 8 | 0.01 | | 3.3 | 0.3 | 0.022 | 1.24 | 1.8 |
| 9 | 0.004 | | 3.3 | 0.1 | 0.013 | 1.24 | 1.0 |
| 10 | 0.003 | | 3.3 | 0.1 | 0.015 | 1.24 | 1.2 |
| 11 | 0.00022 | | 0.68 | 0.03 | 0.002 | 1.24 | 0.16 |
| 12 |  | |  |  | 0.02 | 0.84 | 2.38 |
| 13 |  | |  |  | 0.013 | 0.84 | 1.54 |
| 14 |  | |  |  | 0.012 | 0.84 | 1.43 |
| 15 |  | |  |  | 0.023 | 0.63 | 3.6 |
| 16 |  | |  |  | 0.02 | 2 | 1.1 |
| 17 |  | |  |  | 0.01 | 2 | 0.5 |
| 18 |  | |  |  | 0.007 | 2 | 0.4 |
| 19 |  | |  |  | 0.026 | 2 | 1.4 |
| 20 |  | |  |  | 0.017 | 2 | 0.9 |
| 21 |  | |  |  | 0.003 | 1.9 | 0.2 |
| 22 |  | |  |  | 0.003 | 1.9 | 0.2 |
| 23 |  | |  |  | 0.006 | 1.9 | 0.3 |
| 24 |  | |  |  | 0.004 | 1.9 | 0.2 |
| 25 |  | |  |  | 0.012 | 1.83 | 0.7 |
| 26 |  | |  |  | 0.021 | 1.83 | 1.1 |
| 27 |  | |  |  | 0.018 | 1.83 | 1.0 |
| 28 |  | |  |  | 0.007 | 1.82 | 0.4 |
| 29 |  | |  |  | 0.011 | 1.83 | 0.6 |
| 30 |  | |  |  | 0.016 | 1.83 | 0.9 |
| 31 |  | |  |  | 0.007 | 1.83 | 0.4 |
| **Mean** |  | |  | **1.15** |  |  | **0.52** |
| **St. Deviation** | | | | **0.88** |  |  | **0.49** |
| **St. Error** | |  |  | **0.16** |  |  | **0.15** |
| **Unpaired t-test** | | |  |  |  |  | **P=0.03** |
