# Supplementary material for: Trace elements during primordial plexiform network formation in human cerebral organoids
Source: PeerJ. 2017 Feb 8;5:e2927. doi: 10.7717/peerj.2927 (PMC5301978; doi:10.7717/peerj.2927)
Supplement: Data S3 [file peerj-05-2927-s008.doc]

| **30-days old organoids** | | | | | **45-days old organoids** | | | |
| --- | --- | --- | --- | --- | --- | --- | --- | --- |
| **Sample** | **Organoid slice area (mm^2^)** | **Number of PH3 positive cells** | **PH3 positive cells/mm^2^** | | **Organoid slice area (mm^2^)** | | **Number of PH3 positive cells** | **PH3 positive cells/mm^2^** |
| 1 | 0.16 | 22 | 135.8 | | 1.2 | | 17 | 14.2 |
| 2 | 0.60 | 65 | 107.4 | | 1.3 | | 19 | 14.6 |
| 3 | 0.80 | 86 | 107.8 | | 1.6 | | 43 | 26.3 |
| 4 |  |  |  | | 1.2 | | 36 | 28.8 |
| 5 |  |  |  | | 1.5 | | 25 | 16.2 |
| **Mean** |  | | | **117.0** |  | |  | **20.0** |
| **St. Deviation** | | | | **16.28** |  | |  | **6.98** |
| **St. Error** | | | | **9.4** |  | |  | **3.1** |
| **Unpaired t-test** | | | | | | **P<0.0001** | | |
