# Supplementary material for: Trace elements during primordial plexiform network formation in human cerebral organoids
Source: PeerJ. 2017 Feb 8;5:e2927. doi: 10.7717/peerj.2927 (PMC5301978; doi:10.7717/peerj.2927)
Supplement: Data S4 [file peerj-05-2927-s009.doc]

| **30-days old organoids** | | | | **45-days old organoids** | | |
| --- | --- | --- | --- | --- | --- | --- |
| **Sample** | **Organoid slice area (mm^2^)** | **MAP2 positive area (mm^2^)** | **Percentual of MAP2 positive area** | **Organoid slice area (mm^2^)** | **MAP2 positive area (mm^2^)** | **Percentual of MAP2 positive area** |
| 1 | 0.92 | 0.27 | 28.88 | 0.29 | 0.1 | 34.96 |
| 2 | 0.81 | 0.18 | 22.40 | 0.7 | 0.36 | 51.57 |
| 3 | 0.69 | 0.15 | 22.32 | 1.09 | 0.69 | 62.93 |
| 4 | 0.68 | 0.16 | 23.53 | 2.3 | 2.2 | 95.65 |
| 5 |  |  |  | 0.68 | 0.22 | 31.81 |
| 6 |  |  |  | 0.82 | 0.52 | 63.37 |
| **Mean** |  | | **24.28** |  |  | **56.72** |
| **St. Deviation** | | | **3.11** |  |  | **23.32** |
| **St. Error** |  | | **1.55** |  |  | **9.5** |
| **Unpaired t-test** | | | | **P=0.026** | | |
