# Supplementary material for: Trace elements during primordial plexiform network formation in human cerebral organoids
Source: PeerJ. 2017 Feb 8;5:e2927. doi: 10.7717/peerj.2927 (PMC5301978; doi:10.7717/peerj.2927)
Supplement: Data S5 [file peerj-05-2927-s010.doc]

| **30-days old organoids** | | | | | **45-days old organoids** | | | |
| --- | --- | --- | --- | --- | --- | --- | --- | --- |
| **Sample** | **Organoid slice area (mm^2^)** | **Number of** **GAD67 positive cells** | **GAD67 positive cells/mm^2^** | | **Organoid slice area (mm^2^)** | | **Number of GAD67 positive cells** | **GAD67 positive cells/mm^2^** |
| 1 | 0.51 | 29 | 56.86 | | 2.07 | | 430 | 207.73 |
| 2 | 1.27 | 27 | 21.26 | | 0.7 | | 235 | 336.19 |
| 3 |  |  |  | | 0.68 | | 161 | 237.81 |
| 4 |  |  |  | | 0.98 | | 180 | 183.67 |
| **Mean** |  | | | **39.06** |  | |  | **241.4** |
| **St. Deviation** | | | | **25.2** |  | |  | **67.0** |
| **St. Error** | | | | **17.8** |  | |  | **33.5** |
| **Unpaired t-test** | | | | | | **P=0.017** | | |
