# Supplementary material for: Trace elements during primordial plexiform network formation in human cerebral organoids
Source: PeerJ. 2017 Feb 8;5:e2927. doi: 10.7717/peerj.2927 (PMC5301978; doi:10.7717/peerj.2927)
Supplement: Data S6 — The fluorescence intensity in the cerebral organoids’ border was normalized for the tissue background and was given as fold increase on basal condition. [file peerj-05-2927-s011.doc]

| **30-days old organoids** | | | | | **45-days old organoids** | | | |
| --- | --- | --- | --- | --- | --- | --- | --- | --- |
| **Sample** | **Tissue background (a.u.)** | | **Organoid border (a.u.)** | **Organoid border/**  **Tissue background** | **Tissue background (a.u.)** | | **Organoid border (a.u.)** | **Organoid border/**  **Tissue background** |
| 1 | 14.06 | | 22.75 | 1.62 | 6.86 | | 22.58 | 3.29 |
| 2 | 11.99 | | 15.26 | 1.27 | 3.75 | | 17.27 | 4.60 |
| 3 | 19.49 | | 21.27 | 1.09 | 4.97 | | 24.93 | 5.02 |
| 4 | 26.5 | | 33.88 | 1.28 | 9.36 | | 58.12 | 6.20 |
| 5 | 13.14 | | 35.72 | 2.72 | 22.43 | | 49.15 | 2.19 |
| 6 | 18.32 | | 26.06 | 1.42 | 11.40 | | 33.95 | 2.98 |
| 7 |  | |  |  | 10.59 | | 43.59 | 4.12 |
| **Mean** |  | | | **1.57** |  | |  | **4.06** |
| **St. Deviation** | | | | **0.59** |  | |  | **1.36** |
| **St. Error** | |  | | **0.24** |  | |  | **0.51** |
| **Unpaired t-test** | | | | | | **P=0.0016** | | |
