# Supplementary material for: Trace elements during primordial plexiform network formation in human cerebral organoids
Source: PeerJ. 2017 Feb 8;5:e2927. doi: 10.7717/peerj.2927 (PMC5301978; doi:10.7717/peerj.2927)
Supplement: Data S7 [file peerj-05-2927-s012.docx]

| **Sample** | **Diameter size (μm)** | | | |
| --- | --- | --- | --- | --- |
|  | **Days of differentiation** | | | |
|  | **7 days** | **15 days** | **30 days** | **45 days** |
| 1 | 422 | 594 | 1,370 | 1,654 |
| 2 | 550 | 608 | 1,031 | 1,170 |
| 3 | 521 | 637 | 1,255 | 1,544 |
| 4 | 657 | 605 | 1,071 | 1,210 |
| 5 | 487 | 627 | 1,098 | 1,398 |
| 6 | 440 | 659 | 966 | 1,579 |
| 7 | 471 | 785 | 1,283 | 1,638 |
| 8 | 564 | 643 |  | 1,391 |
| 9 |  | 578 |  | 1,689 |
| 10 |  | 730 |  | 1,352 |
| 11 |  | 693 |  |  |
| 12 |  | 646 |  |  |
| 13 |  | 732 |  |  |
| 14 |  | 771 |  |  |
| 15 |  | 693 |  |  |
| **Mean** | **514** | **666.7** | **1,153** | **1,463** |
| **St. Deviation** | **76.36** | **64.61** | **149.4** | **185.5** |
| **St Error** | **27** | **16.68** | **56.48** | **58.67** |
