# Supplementary material for: Trace elements during primordial plexiform network formation in human cerebral organoids
Source: PeerJ. 2017 Feb 8;5:e2927. doi: 10.7717/peerj.2927 (PMC5301978; doi:10.7717/peerj.2927)
Supplement: Data S8 [file peerj-05-2927-s013.doc]

|  | **30-days old organoids**  **Cell density (number of cells/μm^2^)** | | **45-days old organoids**  **Cell density (number of cells/μm^2^)** | |
| --- | --- | --- | --- | --- |
| **Sample** | **Organoid center** | **Organoid Edge** | **Organoid center** | **Organoid Edge** |
| **1** | 0.0042 | 0.0182 | 0.0148 | 0.0169 |
| **2** | 0.0039 | 0.0187 | 0.0140 | 0.0216 |
| **3** | 0.0037 | 0.0131 | 0.0137 | 0.0188 |
| **4** | 0.0031 | 0.01872 | 0.0193 | 0.0237 |
| **5** |  | 0.0136 | 0.0227 | 0.0225 |
| **6** |  | 0.0139 | 0.0153 | 0.0285 |
| **7** |  | 0.0128 |  |  |
| **8** |  | 0.0147 |  |  |
| **9** |  | 0.0099 |  |  |
| **10** |  | 0.0193 |  |  |
| **11** |  | 0.0146 |  |  |
| **12** |  | 0.0143 |  |  |
| **13** |  | 0.0112 |  |  |
| **14** |  | 0.0102 |  |  |
| **Mean** | **0.0037** | **0.0145** | **0.0166** | **0.0220** |
| **St. Deviation** | **0.0005** | **0.0032** | **0.0036** | **0.0040** |
| **St. Error** | **0.0002** | **0.0008** | **0.0015** | **0.0016** |
| **Unpaired t-test** |  | **P<0.0001** |  | **P=0.035** |
